# Community social capital and suicide mortality in the Netherlands: a cross-sectional registry-based study

**DOI:** 10.1186/1471-2458-13-969

**Published:** 2013-10-18

**Authors:** Anton E Kunst, Carolien van Hooijdonk, Mariël Droomers, Johan P Mackenbach

**Affiliations:** 1 Department of Public Health, Academic Medical Centre (AMC), University of Amsterdam, PO Box 22660, 1100 DD, Amsterdam, The Netherlands; 2 Department of Public Health, Erasmus MC, University Medical Centre Rotterdam, Rotterdam, The Netherlands; 3 National Institute of Public Health and the Environment, Bilthoven, the Netherlands

**Keywords:** Suicide, Mortality, Geography, Social capital, Social cohesion

## Abstract

**Background:**

Evidence on the effect of community social capital on suicide mortality rates is fragmentary and inconsistent. The present study aims to determine whether geographic variations in suicide mortality across the Netherlands were associated with community social capital.

**Methods:**

We included 3507 neighbourhoods with 6207 suicide deaths in the period 1995–2000. For each neighbourhood, we measured perceived social capital using information from interview surveys, and we measured structural aspects of social capital using population registers. Associations with mortality were determined using Poisson regression analysis with control for confounders at individual level (age, sex, marital status, country of origin) and area level (area income, population density, religious orientation).

**Results:**

Suicide mortality rates were related to the measure of perceived social capital. Mortality rates were 8 percent higher (95% confidence interval (CI): 2 to 16 percent) in areas with low capital. In stratified analyses, this difference was found to be significantly larger among men (12 percent, CI: 2 to 22) than women (1 percent, CI: -9 to 13), larger among those age 0–50 (18 percent, CI: 8 to 29) than older residents (−2 percent, CI: -12 to 8), and larger among the unmarried (30 percent, CI: 16–45) than the married (−2 percent, CI: -12 to 9). Associations with the structural aspect of social capital were in the same direction, but weaker, and not statistically significant.

**Conclusions:**

This study contributed some evidence to assume a modest effect of community social capital on suicide mortality rates. This effect may be restricted to specific population groups such as younger unmarried men.

## Background

Community social capital has gained increasingly more popularity as a means through which to improve the mental health of populations at local or national levels [1-4]. Social capital has been defined in various ways, including the well-known definition of Putnam as “those features of social structures (such as levels of interpersonal trust and norms of reciprocity and mutual aid), which act as resources for individuals and facilitate collective action” [5].

In principle, community social capital could directly influence mental health of residents in various ways [6]. High levels of social capital might stimulate collective action and mobilisation of resources, including social and mental health services. For individual residents, living in a high-capital community may foster positive psychosocial mechanisms related to feelings of security, identity, and shared emotional connection. Despite these expectations, there is no consistent evidence for positive effects of community social capital on the mental health of resident populations [7-9].

This also applies to suicide. Ever since the classic studies of Durkheim, large geographical variations in suicide mortality have been observed in many countries [10]. Many studies documented that such variations were related to socioeconomic conditions and social-cultural traditions [11]. Yet, it is uncertain whether these geographical patterns are also related to different levels of community social capital [12]. A review in 2005 identified only two studies that assessed associations between regional suicide mortality rates and measures of social capital - with opposing results [7]. A recent study comparing 11 European countries found that national-level suicide rates were inversely related to social trust, but not clearly related to other indicators of social capital (e.g. helpfulness, fairness, participation in activities, and trust in organizations) [13]. Similarly, a Japanese study comparing 20 administrative municipalities of Tokyo found suicide rates to be inversely related to social trust, but not to other indicators of social capital (fairness, helpfulness, organizational membership, and confidence in organizations) [14].

In a previous paper from the Netherlands, we observed that community social capital was not substantially related to small-area variations in all-cause mortality and most causes of death [15]. However, substantial effects were suggested for suicide mortality. Neighbourhoods with higher level of social capital had about 10 percent lower suicide mortality rates as compared to other neighbourhoods [15]. The purpose of the present paper is to further analyse this preliminary evidence for a protective effect of community social capital. We will extend previous work in three ways.

First, we will use complementary measures of community social capital. The previous paper only applied a measure based on self reports of respondents to survey questions on social relationships [15]. This common measurement approach stresses the perceptive, cognitive and behavioural aspects of social capital. We will also use a measure emphasising structural measures of social capital [7]. Such measures complement perceptive measures because they focus on the objectively measurable conditions that constitute levels of social capital in an area. More specifically, we will use an adaptation of Congdon’s measure of community demographics and social fragmentation [16-18].

Second, we will assess the specific role of community social capital against the background of wider characteristics of neighbourhoods. Geographical variations in social capital depend on historical, social-cultural, and socioeconomic conditions. These conditions could act as confounders if they are independently related to suicide mortality rates. In the Netherlands, particular attention may need to be given to the large social-cultural variations within the country, which traditionally were associated with differences in religious denomination [19].

Third, we will assess cross-level interactions. Geographic studies of suicide mortality observed interactions between area-level variables and characteristics of individuals [12]. For example, a study on suicide rates within London observed a higher proportion of non-white residents in an area to be associated with lower suicide mortality rates among non-whites, but with higher suicide rates among whites [20]. However, cross-level interactions are not observed in each study [21]. Such interactions have, to our knowledge, not been investigated in any previous study on suicide and social capital.

The current paper utilises a national data set representing all suicide deaths in the Netherlands in the period 1996–2000. We will study variations in suicide mortality between 3507 Dutch neighbourhoods in relationship to two indicators of social capital (perceptive/behavioural and structural/demographic) and with control for four background characteristics of these areas (socioeconomic level, population density and two cultural-religious variables). Moreover, four characteristics of individual residents (age, sex, marital status and country of origin) will be used both to control for compositional confounding and to assess cross-level effects.

## Methods

### Mortality data

Mortality records and corresponding population data for the years 1995 through 2000 were provided by Statistics Netherlands (CBS). Mortality records were linked to population registry data using personal identification numbers. Persons could enter (by birth or immigration) or leave the study (by death or emigration) during this period. All persons who died during the study period were registered, irrespective of whether the death occurred in the Netherlands or abroad. Suicide deaths were classified according to the ICD-9 (in 1995, with codes E950-E959) and the ICD-10 (1996–2000, with codes X60-X84).

For each person, information was available on sex, five-year age group, marital status (never married, married, divorced, widowed), country of origin (Dutch, Turkish, Moroccan, Antillean, Surinamese and other non-western) and place of residence (using postcode data). Following the standard definition of CBS, a person was considered to be of non-western origin if at least one of the parents was born in Turkey, Africa, Latin America or Asia (excluding Japan and the former Dutch colony of Indonesia) [22].

Using the postcode information, the place of residence was classified according to a type of neighbourhoods called “buurten” by CBS. These areas were expected to be most meaningful for the study of relationships between suicide and community social capital. On the average, these areas were only about 3.4 km^2^ large and had only 1486 residents. Moreover, most of these areas were demarcated in socioeconomically or geographically meaningful terms, and therefore they were likely to correspond, at least in part, to people’s perceptions of what constitutes their community.

All variables, including place of residence, were measured per January 1 of 1995. Results could thus not be influenced by the occurrence of migration between neighbourhoods during the study period.

### Measurement of neighbourhood characteristics

Neighbourhood data were mostly derived from the Housing Demand Survey of 1998 (WBO). This national survey is based on a randomly drawn sample of the Dutch population aged 18 years and over. Information was available for 117,569 individuals i.e. 0.76% of the Dutch population. Response rates across all municipalities were 51 percent.

WBO data were used to assess the level of social capital within each neighbourhood in terms of the residents’ perception and behaviour. The WBO included 13 questions on aspects of community social capital as defined by Putnam [5]. These items represented topics such as the attachment to the neighbourhood, degree of interaction with neighbours, and responsibility for the neighbourhood environment. Unrotated principal component analysis with correlation matrix was used to identify a mutual component. All thirteen items had a correlation of 0.60 or higher with the first component. The first component explained 54% of all variation, with a Cronbach’s alpha of 0.60. The level of community social capital was calculated as the average score on this component of all residents that participated in the survey. On the basis of this score, neighbourhoods were ranked into three tertiles with each category containing an equal number of Dutch citizens. For further details, including the selection of thirteen items, we refer to our previous work [15].

A complementary measure of social capital focussed on the structural dimension, as measured by demographic variables. For this, we applied an index developed by Congdon [16,17]. This index equals the sum of the percentage of adult residents living alone, the percentage of households headed by single parents, the percentage of residents living less than 1 year in the neighbourhood, and the percentage of households renting a house under social renting schemes. Data on these four indicators were derived from the continuous population registry or the WBO survey. Information on private renting was not available, and therefore excluded from the index. On the basis of this index, neighbourhoods were ranked into tertiles.

Income data were derived from tax registries for the year 1995. From these data, we measured the percentage of residents with an income below the 40 percent level of the national income distribution (i.e. less than 12,025 euro in 1995). This specific income measure was selected because it was most strongly related to neighbourhood variation in mortality across the Netherlands. The inclusion of other income measures did not substantially contribute to the explanation of mortality variations [15]. No information was available on the educational level or occupational class of residents of neighbourhoods.

The population density of neighbourhoods was measured by means of the number of addresses per square kilometre. Data were obtained from population registry for 1995. Application of an alternative measure, based on the population size of municipalities, show relationships similar to those reported in this paper.

The cultural-religious orientation of the area was measured by (a) the proportion of residents who reported to belong to the Roman Catholic Church, and (b) the percentage of residents that went to church or mosque at least once a week. The first measure is potentially relevant to the Netherlands because of the traditional distinction between the Roman Catholic south and the protestant West and North of the country, which is associated with long-standing differences in culture and social life. The latter measure is especially relevant to distinguish between traditional, orthodox areas and secularised, modern, liberal areas [19]. Data on both variables were derived from the WBO survey.

Neighbourhoods with less than 5 respondents in the WBO survey (in total 6,863 neighbourhoods) were excluded from analyses because social capital measures based on such a small sample were imprecise. Sensitivity analyses have found results of the present study to be robust against an alternative cut off point of 20 respondents per neighbourhood. Another 11 neighbourhoods were excluded because of missing data on neighbourhood income level. Analyses were performed on the remaining 3,507 neighbourhoods (33.78% of all neighbourhoods in the Netherlands) with 11,037,640 residents (71.56% of all Dutch citizens). Excluded areas mostly consisted of small rural neighbourhoods and industrial sites.

The selected neighbourhoods were ranked according to their scores on the contextual variables, and grouped into five quintiles (for the background variables) or three tertiles (for the social capital measures) with each category containing an equal number of Dutch citizens.

### Statistical analysis

Log linear regression models assuming Poisson distribution of error terms were used to measure the association between area levels of suicide mortality and contextual determinants. In the regression method, control was made for population size and population composition by means of an offset variable. This offset variable was calculated as the logarithm of the expected number of deaths for each neighbourhood. This expected number was calculated taking into account the distribution of the local population according to sex, age, country of origin and marital status. For details, we refer to our previous publication [15].

Three types of regression models were fitted to make a stepwise adjustment for sex, age, country of origin (model 1), marital status (model 2), and contextual variables (model 3). In models 1 and 2, we adjusted for individual-level variables using the offset variable as specified above. In model 3, contextual variables were added to the regression model as independent variables. Subgroup analyses were performed according to gender (male or female), age (0–49 or 50+ years), marital status (non-married or married) and country of origin (western or non-western).

In addition, we performed statistical tests for the interaction between social capital variables (perceptive or structural) and individual-level variables (sex, age, country of origin, or marital status). For each combination (e.g. perceptive social capital by sex) we added the corresponding interaction term to model 3 as described above, and we assessed the improvement of the fit of the model using likelihood ratio tests. Statistical significance was defined at conventional levels (p-value < 0.05).

Regression coefficients were converted into Rate Ratios by means of log-transformation. For social capital measures, these Rate Ratios represent the mortality in neighbourhoods with low or medium social capital, taking the high capital group as reference. 95 percent confidence intervals were derived from the standard errors to the regression coefficients.

This study has been performed in accordance with the Declaration of Helsinki. The study did not require ethics approval, because it is only based on existing registry-based data, and these data were analyzed with complete adherence to the strict privacy protection rules of Statistics Netherlands.

## Results

An overview of the independent variables is presented in Table 1. Levels of social capital are strongly correlated with low population density (correlation of −0.508). Weaker associations (correlations of about 0.20) are observed with the presence of low income groups and cultural-religious characteristics of areas. The two social capital measures are only weakly correlated (0.218), underlining the potentially added value of the structural, demographic measure.

**Table 1:** Distribution of contextual variables across neighbourhoods in the Netherlands

**Neighbourhood**	**Mean**	**Standard deviation**	**Minimum**	**Maximum**	**Correlation with social capital**
Social capital (perceptive/behavioural)	0.00	1.00	−3.60	4.64	1.000
Social capital (structural/demographic)	0.02	2.32	−4.27	14.28	0.218
Percentage residents with a low income	38.79	7.53	16.00	76.00	−0.241
Population density (no. addresses/km^2^)	1211	1141	16	11330	−0.508
Percentage Roman Catholics	32.92	29.54	0	100	0.150
Percentage residents that go to church or mosque	14.41	10.64	0	85.71	0.209

During the 6-year study period, the average number of suicide deaths per neighbourhood was 1.77, with standard deviation of 2.28, and a maximum of 33 deaths in one large neighbourhood. Suicide mortality levels differed in relationship to most areas characteristics (Table 2). After control for age, sex and country of origin, suicide rates were 60 percent higher in areas with the lowest compared to highest income. This difference reduced to 33 percent after control for marital status. Variations in suicide rates according to population density and cultural-religious variables were smaller and non-linear. Control for these contextual variables hardly affected the relationship between suicide rates and neighbourhood income level.

**Table 2:** Association of suicide rates with background contextual variables

**Contextual variable**		**Number of suicides**	**Rate ratio (95% confidence interval) estimated with control for**					
			**Age, sex, country of birth**		**Plus marital status**		**Plus all background contextual variables**	
Neighbourhood income level	Low	1651	1.60	(1.48–1.73)	1.33	(1.23–1.44)	1.31	(1.21–1.42)
		1370	1.28	(1.18–1.39)	1.18	(1.09–1.28)	1.17	(1.08–1.27)
		1021	1.12	(1.03–1.22)	1.08	(0.99–1.18)	1.07	(0.98–1.17)
		1135	1.03	(0.94–1.12)	1.01	(0.93–1.10)	1.01	(0.93–1.10)
	High	1030	1.00		1.00		1.00	
Population density (no. addresses/km^2^)	Urban	1609	1.45	(1.34–1.56)	1.12	(1.04–1.21)	1.05	(0.96–1.14)
		1273	1.18	(1.09–1.28)	1.05	(0.97–1.14)	1.03	(0.95–1.12)
		1126	1.07	(0.98–1.16)	1.01	(0.93–1.09)	1.01	(0.93–1.10)
		1125	1.06	(0.98–1.16)	1.04	(0.96–1.14)	1.05	(0.97–1.15)
	Rural	1074	1.00		1.00		1.00	
Percentage Roman Catholics	Low	1169	0.96	(0.88–1.05)	0.92	(0.85–1.00)	0.92	(0.84–1.01)
		1235	0.98	(0.91–1.07)	0.92	(0.84–1.00)	0.92	(0.84–1.01)
		1288	1.06	(0.98–1.15)	0.97	(0.89–1.05)	0.99	(0.90–1.07)
		1495	1.04	(0.96–1.12)	0.99	(0.92–1.08)	1.02	(0.94–1.10)
	High	1020	1.00		1.00		1.00	
Percentage residents that go to church or mosque	Low	1390	1.28	(1.18–1.38)	1.12	(1.03–1.21)	1.02	(0.93–1.11)
		1282	1.17	(1.08–1.27)	1.11	(1.03–1.21)	1.06	(0.97–1.16)
		1276	1.15	(1.06–1.25)	1.07	(0.99–1.16)	1.03	(0.95–1.12)
		1192	1.11	(1.02–1.21)	1.04	(0.96–1.13)	1.02	(0.94–1.11)
	High	1067	1.00		1.00		1.00	

Results of multivariate regression analyses.

Suicide mortality rates were 30% higher in areas with low community social capital according to the perceptive/behavioural measure (Table 3). Half of this difference could be explained by control for marital status at the individual level, and an additional part could be explained by control for area characteristics. An 8 percent difference remained in the full model, with 95 percent confidence ranging from 2 to 16 percent. Associations with the structural/demographic measure of social capital were weaker, and in the final model not statistically significant.

**Table 3:** Association of suicide rates with the two measures of community social capital

**Contextual variable**		**Number of suicides**	**Rate ratio (95% confidence interval) estimated with control for**					
			**Age, sex, country of birth**		**Plus marital status**		**Plus all background contextual variables**	
Social capital (perceptive/behavioural)	Low	2434	1.31	(1.24–1.40)	1.15	(1.08–1.22)	1.08	(1.02–1.16)
	Medium	1902	1.02	(0.96–1.09)	0.98	(0.92–1.04)	0.98	(0.92–1.05)
	High	1871	1.00		1.00		1.00	
Social capital (structural/demographic)	High	2254	1.22	(1.15–1.30)	1.08	(1.02–1.15)	1.02	(0.96–1.09)
	Medium	2014	1.10	(1.04–1.18)	1.05	(0.99–1.12)	1.03	(0.97–1.10)
	Low	1939	1.00		1.00		1.00	

Results of multivariate regression analyses.

Results of stratified analyses are presented in Table 4. The perceptive/behavioural measure of social capital was associated with a 12 percent difference in suicide mortality among men, compared to only a 1 percent difference among women. Similarly, an 18 percent mortality difference is observed for the age group 0–50 years, compared to no differences among older age groups. A 30 percent difference is observed among the non-married, compared to no differences among the married. Statistical tests showed that the interactions with gender, age and marital status were statistically significant (p-value < 0.05). The interaction with country of origin was statistically significant, but small in substantive terms. Interactions with the structural/demographic measure of social capital were weaker though in the same direction. None of these latter interactions were statistically significant after full control (p-value > 0.05).

**Table 4:** Association between social features of the neighbourhood and suicide according to social-demographic characteristics of residents

		**Rate ratio (95% confidence interval)**							
***A. Stratification by sex***									
		**Men**				**Women**			
		**Control for age, sex, country origin, marital status**		**Plus control for background contextual variables**		**Control for age, sex, country origin, marital status**		**Plus control for background contextual variables**	
Social capital (perceptive/ behavioural)	Low	1.17	(1.09–1.26)	1.12	(1.04–1.22)	1.11	(1.00–1.23)	1.01	(0.91–1.13)
	Medium	1.00	(0.93–1.08)	1.01	(0.94–1.10)	0.93	(0.84–1.04)	0.92	(0.83–1.03)
	High	1.00	--	1.00	--	1.00	--	1.00	--
Social capital (structural/ demographic)	High	1.09	(1.01–1.17)	1.04	(0.96–1.12)	1.07	(0.97–1.19)	0.98	(0.88–1.10)
	Medium	1.05	(0.97–1.13)	1.03	(0.95–1.11)	1.07	(0.96–1.19)	1.04	(0.93–1.16)
	Low	1.00	--	1.00	--	1.00	--	1.00	--

***B. Stratification by age***									
		**0-50 years olds**				**50 years and older**			
		**Control for age, sex, country origin, marital status**		**Plus control for background contextual variables**		**Control for age, sex, country origin, marital status**		**Plus control for background contextual variables**	
Social capital (perceptive/ behavioural)	Low	1.26	(1.16–1.37)	1.18	(1.08–1.29)	1.01	(0.92–1.11)	0.98	(0.88–1.08)
	Medium	1.04	(0.96–1.14)	1.05	(0.96–1.15)	0.91	(0.82–1.00)	0.91	(0.82–1.00)
	High	1.00	--	1.00	--	1.00	--	1.00	--
Social capital (structural/ demographic)	High	1.15	(1.06–1.24)	1.05	(0.96–1.14)	1.00	(0.91–1.10)	0.98	(0.89–1.08)
	Medium	1.08	(1.00–1.18)	1.05	(0.97–1.15)	1.02	(0.93–1.12)	1.02	(0.92–1.12)
	Low	1.00	--	1.00	--	1.00	--	1.00	--

***C. Stratification by marital status***									
		**Unmarried**				**Married**			
		**Control for age, sex, country origin, marital status**		**Plus control for background contextual variables**		**Control for age, sex, country origin, marital status**		**Plus control for background contextual variables**	
Social capital (perceptive/ behavioural)	Low	1.39	(1.26–1.54)	1.30	(1.16–1.45)	1.00	(0.91–1.11)	0.98	(0.88–1.09)
	Medium	1.10	(0.99–1.23)	1.10	(0.98–1.23)	0.89	(0.80–0.98)	0.90	(0.81–1.00)
	High	1.00	--	1.00	--	1.00	--	1.00	--
Social capital (structural/ demographic)	High	1.19	(1.08–1.32)	1.07	(0.96–1.19)	1.04	(0.94–1.15)	1.01	(0.91–1.13)
	Medium	1.11	(1.00–1.24)	1.08	(0.97–1.20)	1.07	(0.97–1.18)	1.07	(0.97–1.18)
	Low	1.00	--	1.00	--	1.00	--	1.00	--

***D. Stratification by country of origin***									
		**Western**				**Non-western**			
		**Control for age, sex, country origin, marital status**		**Plus control for background contextual variables**		**Control for age, sex, country origin, marital status**		**Plus control for background contextual variables**	
Social capital (perceptive/ behavioural)	Low	1.16	(1.08–1.23)	1.09	(1.01–1.17)	1.19	(1.01–1.40)	1.10	(0.92–1.31)
	Medium	0.97	(0.90–1.03)	0.97	(0.90–1.04)	1.08	(0.90–1.29)	1.07	(0.89–1.29)
	High	1.00	--	1.00	--	1.00	--	1.00	--
Social capital (structural/ demographic)	High	1.09	(1.02–1.16)	1.02	(0.95–1.09)	1.10	(0.94–1.29)	1.04	(0.88–1.23)
	Medium	1.04	(0.97–1.11)	1.02	(0.95–1.09)	1.15	(0.98–1.36)	1.12	(0.94–1.32)
	Low	1.00	--	1.00	--	1.00	--	1.00	--

## Discussion

The importance of social integration to suicide has been emphasised ever since Durkheim [10]. An early study on the Netherlands in the early 20^th^ century observed that suicide mortality rates were high in small, isolated villages where social life and moral climate were dominated by a gloomy form of orthodox Protestantism [23]. Suicide rates were particularly high in municipalities with social disintegration, as indicated by a high percentage of voters on fascist parties [23].

Our study follows this early example by assessing, for the end of the 20^th^ century, the relationship with the modern concept of community social capital. We observed large differences in suicide mortality in relation to two measures of community social capital. About one half of these differences could be explained by the compositional effect of marital status. Control for background contextual variables, especially local income levels, explained an additional part of these differences. The residual differences were modest for the total population (8 percent) but demonstrably larger for specific groups of residents (men, those younger than 50 years, and non-married people).

### Evaluation of strengths and limitations

Our data covered small areas across the entire country. This also implied a methodological difference to those studies that were restricted to cities or metropolitan areas. Further analysis showed that we would have observed slightly larger effects of community social capital on suicide mortality rates if we were to restrict the analysis to the four largest cities of the Netherlands (*results not shown*).

We had to restrict the analysis to 34 percent of the areas, thus including 72 percent of the total Dutch population. We excluded areas with small numbers of inhabitants, most of which were rural areas. Given the fact that we observed the association between suicide and social capital to be stronger within urban areas than within rural areas (see above), we expect that we would have observed weaker associations if we were to include all rural areas.

We deliberately selected neighbourhoods (“buurten”) that were relatively small and homogeneous. In the Dutch context, these areas were expected to be most meaningful for the postulated causal mechanisms linking social capital to suicide [1-3]. Nonetheless, we may have missed associations that could have been formed at lower geographical scales such as blocks or streets, or at higher scales such as cities or states [13,24].

We identified suicide deaths using ICD codes available in the national cause-of-death registry, which is utilises information available from death certificates. The coverage of this source of information was evaluated by comparison with policy registries. A small degree of underreporting of suicide deaths had been observed, although exact estimates are lacking [25]. However, we do not expect the degree of underreporting to systematically vary between different types of areas, such as areas with different levels of community social capital [19].

We applied a single-level analyses that disregarded the clustering of individuals within neighbourhoods. A multilevel analysis would have been the most appropriate method given the nested structure of the data. However, by the time of our study, a national dataset of deaths nested into 3500 neighbourhoods was too large for running multilevel analyses programs within the CBS infrastructure. Moreover, datasets could not be transferred to computers outside the CBS infrastructure due to data protection rules. Because of disregarding the clustering of individuals within neighbourhoods, estimates of 95 percent confidence intervals should be interpreted with caution as they might be too narrow. However, in recent nation-wide analyses of neighbourhood differences of other health outcomes, we found 95% confidence intervals to be about equally wide in both single-level and multilevel analyses [26,27].

Our key measure of social capital was constructed on the basis of 13 items that were selected from a larger range of potentially relevant questions in the WBO survey. The selection and processing of these items was guided by Putnam’s definition of community social capital [15]. Despite this careful construction, we cannot exclude the possibility that areas have been misclassified in terms of community social capital. If misclassification would be unrelated to suicide mortality rates, it would have resulted in an underestimation of the associations reported in this paper.

### Interpretation of observed patterns

The observed association between community social capital and suicide could for about 50 percent be attributed to the compositional effect of marital status. This underlines the potential effect of population composition on observed associations between community social capital and suicide. Strong compositional effects have also been observed in studies on mental health [12,21]. In our study, the true contribution of compositional effects is likely to exceed the 50 percent, because some unmeasured factors may be important as well. Unmeasured compositional factors include:

1. Household composition. In addition to marital status, factors such as partnership and parenthood have been found to be related to suicide mortality at the individual level [28-30]. These factors are likely to also contribute to area variations in mental health outcomes. For example, one study observed that the association between community social capital and the prevalence of depression could in part be explained by the composition of the population in terms of partnership and parenthood [7].

2. Socioeconomic factors. In our analysis, a quarter of the association between community social capital and suicide rates was explained by variations between areas in an aggregate income measure (Table 3). A considerably larger effect might have been observed if we would have been able to control for individual-level socioeconomic factors. Analyses from Finland and Denmark found that area-level associations between suicide mortality and socioeconomic indicators could almost entirely be explained by individual-level associations [12,21]. Residual confounding by individual-level socioeconomic factors might explain why associations were observed especially for men and for unmarried people. In most European countries, socioeconomic position as measured by educational level has a greater impact on suicide mortality among men compared to women [31] and among non-married compared to married people [32].

3. Social-cultural characteristics of areas. In our analysis, we included religious variables that reflected long-standing socio-cultural variations between Dutch regions [19]. However, religious variables may also operate at the individual level. Suicide risk has found to be related to individual-level religious denomination (including nowadays Islam), church or mosque attendance, and degree of religiosity [33,34]. In as far as the composition of population in such social-cultural terms is related to social capital, this may have confounded the associations of community social capital with suicide risk.

Compositional factors may be associated with community social capital in two distinct ways [12]. On one hand, community social capital may influence population composition. For example, families with young children may be more likely to move to areas with high social capital because of the greater protection that these areas offer to children [35]. On the other hand, the population composition may affect the amount of social capital within an area. For example, the presence of many families with young children may increase interaction among neighbours and feelings of responsibility for the neighbourhood. Conversely, neighbourhood social cohesiveness may be undermined by demographic or ethnic heterogeneity, and by large socioeconomic inequalities [36,37].

The second explanation is part of the selective migration hypothesis. As with other health problems [38,39], people at the greatest risk for suicide are likely to move into, or stay in, neighbourhoods with low community social capital. As a result, neighbourhoods with high in-migration rates may have increased suicide rates [40]. The association between community social capital and suicide mortality might therefore in part be due to selective migration. Consistent with this explanation is our finding of an association among residents younger than 50 years but not among older residents. In the Netherlands, rates of inter-neighbourhood migration are by far higher at ages under 50 years than at older ages [41].

In principle, different population groups may benefit from community social capital to a different extent. We found an association between community social capital and suicide risk only among men, among those aged 0–50, and among the non-married. Could we expect cross-level interactions to have this specific pattern? In theory, one might perhaps expect the largest effects to occur among people for whom the neighbourhood is a more important source of social support. One Dutch study found that residents with small children were most likely to receive support from their neighbours, whereas no large differences were observed by gender or by age [42]. Another Dutch study observed no large differences by age or gender in the relative frequency of contacts with neighbours [43]. Thus, in the Dutch context, there seems no reason to expect community social capital to be most important for men, the young or the unmarried.

## Conclusions

We conclude that, at the end of the 20^th^ century, areas with a high level of community social capital had lower suicide mortality rates. This association is consistent with the view that high levels of community social capital could protect residents against suicide death. However, these associations need to be interpreted with caution. We had no a priori reasons to expect protective effects to be limited to men, the young, or the unmarried. Moreover, the observed associations might also be explained with reference to compositional effects and selective migration processes.

Studies in other countries may assess whether effects of community social capital, if any, are restricted to specific groups such as younger unmarried men. Such studies may benefit from a broader perspective of the social environmental context of suicide, and also assess factors such as social disorder and local cultures. Potential effects may be detected with greater accuracy by studying how such factors influence suicide risk among specific groups of residents.

## Competing interests

The authors declare that they have no competing interests.

## Authors’ contributions

AK and CH conceived the idea for this study. CH carried out the analyses. AK drafted the manuscript. MD and JM participated in study design. CH, MD and JM commented on drafts of the manuscript. All authors read and approved the final manuscript.

## Pre-publication history

The pre-publication history for this paper can be accessed here:

http://www.biomedcentral.com/1471-2458/13/969/prepub
